# Bridging Gaps in Women’s Heart Health: User-Centered Needs Assessment Informed by Patient and Clinician Interviews

**DOI:** 10.2196/82916

**Published:** 2026-01-13

**Authors:** Christine Jacob, Sangeetha-Rose Puthanveettil, Patrick Vavken, Emel Kaplan, Christine S Zuern

**Affiliations:** 1 Institute for Information Systems University of Applied Sciences Northwestern Switzerland (FHNW) Windisch, Aargau Switzerland; 2 University of Applied Sciences Northwestern Switzerland FHNW Windisch Switzerland; 3 Vavken Health Labs Zurich Switzerland; 4 ETH Zurich Switzerland; 5 University of St. Gallen St. Gallen Switzerland; 6 Cardiovascular Research Institute Basel (CRIB) and Department of Cardiology, University Hospital Basel, University of Basel Basel Switzerland

**Keywords:** eHealth, mobile health, mHealth, digital health, user research, technology adoption, technology implementation, qualitative study, usability, cardiovascular disease, women's health, human centered design

## Abstract

**Background:**

Women with cardiovascular disease (CVD) remain underserved due to gaps in recognition, diagnosis, and care tailored to sex-specific risks. Digital health tools have the potential to address these inequities, but many fail to reflect the distinct needs of women. In a prior review, we assessed 20 CVD apps and 22 wearables and found that only 25% (5/20) of apps and 40% (9/22) of wearables included any sex-specific content, such as hormone cycle tracking and life-stage considerations related to pregnancy or menopause. These findings confirm that current digital tools largely mirror the gender gaps seen in traditional care.

**Objective:**

This study aimed to define the user requirements for a CVD app designed specifically for women. We sought to explore the unmet needs and challenges faced by female patients and their clinicians that current tools fail to address, and also to identify and prioritize features that would be most valuable and feasible to implement.

**Methods:**

We conducted a qualitative study using semistructured interviews to explore the needs, preferences, and expectations of women living with CVD and their treating clinicians. Guided by the human-centered design framework, this work focused on the “Define” phase. A total of 20 participants in Switzerland were interviewed, including 11 women with CVD, 7 cardiologists, and 2 experts in regulatory and reimbursement. Participants were recruited through purposive sampling, and interviews were conducted online between April and July 2025. Thematic analysis was used to synthesize the data, highlighting design priorities and contextual factors relevant for developing a patient-centered and system-aware digital health tool.

**Results:**

The interviews with women living with CVD and cardiologists confirmed the consistent gaps between existing care pathways and the specific needs of female patients. Both groups highlighted the lack of early symptom recognition, insufficient sex-specific guidance, and limited tools tailored to women’s lived experience. While patients prioritized personalized education, emotional support, and features that address hormonal and life-stage–specific risks, clinicians emphasized clinical use, workload integration, and actionable summaries. Success was defined experientially by patients (eg, empowerment and reduced anxiety), and operationally by clinicians (eg, earlier detection and improved adherence). Willingness to pay was moderate among both groups, with patients favoring simplicity and clinicians emphasizing workflow integration and proven clinical use.

**Conclusions:**

These findings highlight the importance of designing an artificial intelligence–enabled CVD app for women that meaningfully integrates patient empowerment with clinical workflows. A dual-value approach is essential, offering personalized tools that address emotional and lifestyle needs for patients, while supporting clinicians with concise, actionable insights. Early reflections on regulatory and reimbursement considerations suggest that a modular, evidence-based rollout strategy would be key for long-term adoption and scale.

## Introduction

### Background

Cardiovascular disease (CVD) remains the world’s leading cause of death, claiming nearly 18 million lives each year and impacting more than half a billion people globally [1-3]. Despite decades of progress in cardiology, these numbers underscore a persistent challenge: our current approaches to prevention, diagnosis, and treatment are not reaching everyone equally [3]. One critical blind spot is the under-recognition of sex-based physiological differences in cardiovascular health [4]. Women, in particular, are often underdiagnosed, undertreated, and underserved, partly due to atypical symptom presentation and a longstanding male-centric model of research and care [4]. This has serious consequences, including delays in diagnosis and poorer outcomes for women across the CVD continuum [5].

Digital health technologies offer a promising avenue to change this narrative. Mobile health (mHealth) tools, remote patient monitoring, wearable sensors, and artificial intelligence (AI)–driven decision support systems are reshaping how individuals manage their cardiovascular risk, bringing prevention, detection, and self-care into everyday life [1]. According to the World Health Organization, mHealth refers broadly to health care and public health services supported by mobile devices such as smartphones, wearables, and wireless sensors [6]. These tools can improve access to timely care, enable more tailored interventions, and potentially reduce long-term costs to the health system [7].

Evidence is steadily accumulating in favor of mHealth for cardiovascular care. For example, a systematic review by Coorey et al [8] found that mobile apps can support better blood pressure (BP) control, encourage healthy dietary habits, and reduce hospital readmissions for patients with CVD. Smartphone-based photoplethysmography, a technology that measures changes in blood volume using infrared light, has shown promise in detecting atrial fibrillation (AF) and assessing heart rate (HR) variability, offering an accessible, noninvasive, and scalable solution for early risk detection [9].

Wearable devices are also gaining ground as tools for continuous cardiovascular monitoring. Positioned on the wrist, chest, or hip, these devices can monitor HR, blood oxygen levels, sleep patterns, and physical activity using either photoplethysmography or ECG technology [10,11]. For instance, a study by Guo et al [12] involving more than 187,000 users identified more than 260,000 potential AF episodes, with confirmatory testing validating the diagnosis in most cases. A broader review of smartwatch-based interventions echoed these findings, highlighting improvements in lifestyle behaviors, medication adherence, AF detection, and reductions in unplanned hospitalizations [13].

### Current State of CVD Apps and Wearables

Building on the well-documented unmet needs in CVD for women, we evaluated how effectively existing digital health tools, specifically mobile apps and wearable devices, address sex-specific factors in CVD. To do this, we conducted a structured review of 20 patient-facing CVD apps and 22 commercially available wearables. Each tool was assessed using the foundational and contextual dimensions of the sociotechnical framework for evaluating patient-facing eHealth interventions [14], which emphasizes both technical functionality and integration into real-world health contexts.

The results of this assessment, published in a separate study [15], revealed a significant gap: only 25% (5/20) of the reviewed apps and 40% (9/22) of the wearables incorporated sex-specific content. This included considerations such as the impact of hormonal changes, menopause, or pregnancy on cardiovascular health, factors known to influence symptom presentation, disease progression, and treatment needs in women. These findings reinforce the conclusion that digital health tools are not exempt from the systemic gaps that characterize traditional CVD care pathways [15]. Rather, they mirror the underrepresentation of women’s needs in CVD. Addressing this gap is critical if we aim to develop digital interventions that are both equitable and clinically effective for women living with CVD.

### Objectives

To address this gap, this study explored the user needs and requirements for a digital health app designed specifically for women with CVD. The primary objectives were (1) to understand the specific challenges and unmet needs that female patients and health care professionals encounter in cardiovascular care that are not adequately addressed by current digital tools, and (2) to identify and prioritize the features and functionalities that a new app should incorporate to better support sex-specific cardiovascular health management. Furthermore, the study did not only focus on the preferences and expectations of the primary users, such as patients and clinicians, but also considered broader system-level enablers and constraints, including clinical integration, regulatory requirements, and reimbursement considerations, to guide a practical and scalable development strategy.

## Methods

### Overview

We adopted a qualitative research approach to explore the nuanced needs and expectations of both patients and clinicians. This methodology was chosen for its strength in capturing complex, context-dependent experiences and sociocultural factors that are often overlooked by quantitative methods [16].

Qualitative methods are increasingly used in health services and technology research, as they allow researchers to uncover the “why” behind user behaviors and preferences [17]. In our case, this approach helped surface the specific ways in which digital tools can support women across the CVD journey, and how clinicians view their potential integration into care pathways. These insights provide a strong foundation for user-centered feature development and iterative design.

### Scope and Conceptual Framework

This study was guided by the human-centered design (HCD) framework, which places the needs, experiences, and preferences of end users, here, women living with CVD and the clinicians who care for them, at the core of innovation [18-20]. HCD is a structured, iterative methodology that unfolds across 4 key phases: discover, define, design and prototype, and implement. Each phase plays a critical role in ensuring that health technologies are developed not only for users, but with them [21,22].

The discover phase, which focuses on identifying the problem space and understanding unmet needs, was addressed in our previously published study that assessed the extent to which existing CVD apps and wearables account for sex-specific considerations [15]. That foundational work revealed significant gaps in digital tools for women with CVD, particularly around life-stage–specific guidance and personalized risk tracking [15].

Building on those findings, this study focused on the define phase. This stage aimed to deepen understanding of user needs through qualitative inquiry, synthesize priorities across user groups, and translate insights into clear design criteria. Specifically, we investigated the expectations, preferences, and contextual considerations of both female patients and clinicians to guide the conceptualization of a CVD app tailored to women’s unique health trajectories.

**Figure 1:**
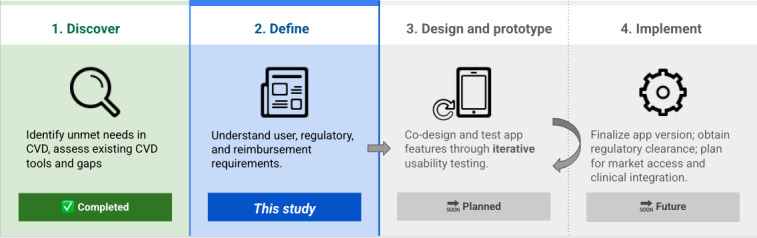
Human-centered design approach for developing an app for women with cardiovascular disease. CVD: cardiovascular disease.

The subsequent design and prototype and implementation phases, where iterative development, user testing, and real-world deployment take place, are beyond the scope of this study but will build directly on the requirements defined in this study. Figure 1 illustrates the HCD process guiding this study, with this study focusing specifically on the define phase, following earlier gap analysis in the discover phase.

### Sampling Strategy and Participant Recruitment

We used purposive sampling, a commonly used strategy in qualitative research aimed at capturing rich, experience-based insights from relevant stakeholders [23]. Patient participants were recruited in collaboration with the Women’s Heart Health Program at the University Hospital Basel, a specialized cardiology outpatient program focusing on women's cardiovascular health. Inclusion criteria required participants to be women aged 18 years or older, have a confirmed diagnosis of CVD, access to email and the internet, and be comfortable using a teleconferencing tool.

Eligible patients were approached directly by the clinical staff in the Women’s Heart Health Program, who explained the study and obtained written informed consent. Once consent was given, the signed forms and contact details were forwarded to the core research team at the University of Applied Sciences and Arts Northwestern Switzerland, who subsequently managed the coordination and communication with participants.

Cardiologists were recruited independently by the research team through targeted online searches and professional outreach via email. The participating cardiologists were recruited from 5 different hospitals and clinics, reflecting 3 care settings across Switzerland, including university hospitals, cantonal hospitals, and private practices, offering a diverse clinical perspective. And unlike clinical or patient experience, which can vary widely, regulatory and reimbursement requirements are governed by defined legal frameworks. Therefore, expert input was sought from two highly specialized professionals with national and EU (European Union)-level expertise, who provided focused guidance on certification and financing pathways for digital health tools in Switzerland and Europe.

All participants received study information and consent materials in both English and German and were given the option to choose their preferred language. Interview duration ranged from 28 to 63 minutes, depending on the depth of participant responses. Recruitment took place between April and June 2025 and continued until thematic saturation was reached, that is, when no new themes emerged from additional interviews [23,24]. This was assessed continuously during data collection and analysis. For both patient and clinician interviews, saturation was monitored separately. We noted that by the tenth patient and sixth clinician interview, no substantially new themes were identified, and the final interviews largely confirmed and elaborated on existing patterns. The English version of the participant information sheet and consent form is included in Multimedia Appendix 1.

A total of 20 participants based in Switzerland were recruited: 11 women living with CVD, 7 cardiologists, 1 regulatory expert, and 1 reimbursement expert. The sample characteristics of the participating patients and clinicians are summarized in Multimedia Appendix 2.

### Data Collection and Synthesis

Data were collected through in-depth, semistructured interviews conducted online between April and July 2025. In total, 2 tailored interview guides were developed: 1 focused on the patient experience and day-to-day disease management, and 1 directed at clinicians, emphasizing workflow, system integration, and implementation considerations. Both guides are provided in Multimedia Appendix 3.

A structured thematic analysis was conducted following the framework by Braun and Clarke [25,26], including familiarization with the data, initial coding, theme development, review, refinement, and final synthesis (see Multimedia Appendix 4 for detailed steps). We used a hybrid coding approach, combining deductive codes informed by prior work and study objectives, with inductive codes that emerged from the data, as described in Multimedia Appendix 4. Codes were grouped into broader thematic categories and, where relevant, mapped to stages of the patient journey (eg, early diagnosis support, treatment, and long-term management). Coding was supported using NVivo (Lumivero) qualitative data analysis software. Patient and clinician interviews were conducted and primarily analyzed by SP, while CJ and PV led the interviews with the regulatory and reimbursement experts. CJ further refined the thematic analysis, synthesis, and reporting. To mitigate potential researcher bias, coding was triangulated across the 3 coauthors (SRP, CJ, and PV), and discrepancies were resolved through iterative discussion. Any coding discrepancies between SP and CJ were addressed through discussions with PV until a consensus was reached. This process spanned from May to August 2025.

### Ethical Considerations

The Ethics Committee of Northwest and Central Switzerland determined that ethics approval was not needed for this study, according to the Federal Act on Research involving Human Beings, article 2, paragraph 1 (reference number Req-2025-00491). All participants were briefed about the research background and signed a consent form agreeing to participate. Participant data were anonymized, access to identifiable source files was restricted to the first and second authors, and no participants received financial compensation, with the exception of the regulatory and market access experts who received consulting fees for their professional advisory contributions.

## Results

### Understanding the User Journey and Unmet Needs

The patient interviews revealed a consistent pattern of late recognition, emotional burden, and lack of gender-sensitive care in the management of CVD. Many women described experiencing a delayed or unclear diagnosis, often attributed to atypical symptom presentation and a lack of accessible, sex-specific information. Many had to self-educate or navigate their condition alone, especially at the outset, before a formal diagnosis was made. Emotional distress, stress, and fear, especially post diagnosis, were prominent themes. Participants spoke about the emotional toll that followed their diagnosis, including feelings of shock, sadness, fear, and uncertainty about the future. For some, the psychological burden was compounded by life-altering restrictions, such as being advised against future pregnancies or having to give up previously enjoyed activities like vigorous exercise.

There was a widespread perception among the participants that hormonal and life-stage influences (eg, pregnancy and menopause) are underacknowledged in treatment pathways. Only 18% (2/11) of patients received any sex-specific guidance, mostly about the potential dangers of getting pregnant with heart disease. Participating patients stated that they generally struggle with lifestyle adjustments, medication adherence, and managing comorbidities. When discussing the complexity of symptom management, participants frequently described difficulties in understanding medical instructions and uncertainty about how to respond to different symptoms or side effects. This was further complicated by external factors such as stress, an acknowledged trigger that patients knew could worsen their condition, yet felt largely powerless to control.

Digital tools are underused; only 18% (2/11) of patients had experience using CVD apps or wearables, for example, for ECG measurement, while the others expressed confusion, lack of awareness, or anxiety about using them. All participating patients agreed that gender-specific tracking would be beneficial. Table 1 highlights the main challenges and unmet needs faced by women with CVD along the care continuum, their current use of digital health tools, and how many participants referenced each theme in the interviews.

**Table 1:** Understanding the patient journey and unmet needs (n=11).

Themes and subthemes		Prevalence, n (%)
**Early symptoms and diagnosis**		
	Unfamiliar with the disease at diagnosis	7 (64)
	Atypical or no symptoms prior to diagnosis	6 (55)
	Self-initiated education (eg, googling and support groups)	5 (45)
	Emotional distress after diagnosis (shock, sadness, and fear)	6 (55)
	Stress and psychological burden of the disease	5 (45)
	CVD^a^ perceived as a burden or life-changing event	6 (55)
**Life stage and gender-specific care**		
	Not informed or addressed by HCPs^b^	9 (82)
	Was informed about the impact of life stages (eg, pregnancy and menopause)	2 (18)
	Perceived treatment as male-centered	4 (36)
	Belief that women’s CVD symptoms are often dismissed	3 (27)
**Ongoing disease management challenges**		
	Difficulty with lifestyle and diet changes	6 (55)
	Struggles with medication adherence or side effects	5 (55)
	Difficulty understanding symptoms and instructions	4 (36)
	Difficulty managing comorbidities	3 (27)
	Anxiety before appointments because of uncertainties (eg, on potential progression)	2 (18)
	Dealing with constant emotional overwhelm and fatigue	3 (27)
**Use of existing digital tools**		
	Has not used any apps for CVD	9 (82)
	Not aware of any helpful CVD apps	7 (64)
	Prefers face-to-face care over digital tools	4 (36)
	Feels overwhelmed or anxious by the idea of apps	3 (27)
	Has used a step counter, hydration reminder, or menstrual tracker	3 (27)
	Has used a CVD-related app or wearable (eg, for ECG^c^ measurement)	2 (18)

^a^CVD: cardiovascular disease.

^b^HCP: health care professional.

^c^ECG: electrocardiogram.

Clinicians reported several diagnostic challenges stemming from atypical symptom presentation in women, compounded by limited awareness and insufficient referral pathways. They emphasized the mismatch between traditional risk models and real-world female presentations, which are often underrecognized or misattributed. Time constraints and health care system overload further exacerbate diagnostic delays. Despite acknowledging the lack of tools to capture hormonal or life-stage influences, 5 of 7 (71%) participating clinicians reported that they continue to follow standard monitoring protocols without sex-specific adjustments.

Only 1 of 7 (17%) interviewed cardiologists recommended CVD apps, while 6 of 7 (86%) cited lack of familiarity or trust in their use, with usability for older patients as the main concern. Integration of digital tools into clinical workflows remains controversial; some see potential for structured summaries and AI-assisted risk insights, while others worry about time burden or data security. There was strong agreement on the need for decision support tools that surface earlier warnings during high-risk windows such as menopause, pregnancy, or postevent follow-ups, with 5 of 7 (71%) participants citing this as an existing gap. Table 2 presents the key challenges and unmet needs encountered by clinicians throughout the clinical workflow, alongside their current engagement with digital health tools, and how many participants referenced each theme in the interviews.

**Table 2:** Understanding the clinician workflow and unmet needs (n=7).

Themes and subthemes		Prevalence, n (%)
**Diagnostic challenges**		
	Atypical symptoms in women make the diagnosis more challenging	7 (100)
	Lack of awareness and, accordingly, referrals	5 (71)
	Limited time and capacity in care	3 (43)
**Sex-specific gaps**		
	Symptoms misattributed or dismissed	7 (100)
	Treatment paths not sex-sensitive and mostly male-focused	5 (71)
**Monitoring practices**		
	Standardized tests, no sex differences	5 (71)
	Limited tools for life-stage tracking in women (eg, pregnancy and menopause)	3 (43)
**Workflow integration needs**		
	Desire for structured reports	6 (86)
	Need decision support during diagnosis and follow-ups	5 (71)
	EHR^a^ integration	3 (43)
**Use of existing digital tools**		
	Does not recommend CVD^b^ apps to their patients	6 (86)
	Low awareness of validated apps	6 (86)
	Too many apps with low usability for older adults	5 (71)

^a^EHR: electronic health record.

^b^CVD: cardiovascular disease.

### Desired Features and Functionality

Based on the patient interviews, several key themes emerged regarding desired features and functionalities for a CVD app tailored to women. Patients emphasized the importance of integrated support across the full care continuum, from early symptom awareness and diagnosis to daily management and communication with health care providers.

Core priorities included the ability to track vital signs, log symptoms, and receive tailored educational content specific to women’s cardiovascular health. Medication reminders, stress management features, and dietary guidance were commonly requested. Also, 9 of 11 (82%) patients expressed interest in syncing the app with wearables to streamline data collection and support longitudinal tracking. Communication features, such as automated report generation and the ability to share real-time data with health care providers, were considered highly valuable, with 8 of 11 (73%) patients supporting real-time feedback or alerts when symptoms are concerning. Importantly, all participants favored sex-specific, personalized recommendations, but some voiced concerns about privacy, complexity, and the risk of being overwhelmed by notifications. Table 3 summarizes the key features and functionalities patients desire in a digital health solution, along with the main barriers and concerns, and indicates how many participants raised each point during the interviews.

Based on the interviews with cardiologists, several priorities emerged. The overarching emphasis was on improving diagnostic precision, treatment adherence, and patient-provider communication, while minimizing time burden and clinical noise. Clinicians expressed strong interest in receiving structured patient-generated data (eg, BP, symptom trends, medication adherence, and hormonal cycle data), especially when aggregated into concise, longitudinal reports. There was broad consensus (7/7, 100%) on the value of sex-specific insights (eg, menopause and pregnancy risks), particularly if tailored to life stages and actionable. However, real-time monitoring or alerts received more mixed responses. Most clinicians (5/7, 71%) preferred periodic summaries over continuous alerts, citing concerns around workload, liability, and alert fatigue.

Communication through the app was not favored by most clinicians, who instead preferred to retain current channels such as email, phone, or in-person visits. While 6 out of 7 (86%) found the ability to customize patient goals important, they highlighted that this must not increase cognitive or administrative burden. Concerns were raised about data quality, patient over-reliance on technology, and integration with existing clinical infrastructure (eg, electronic health records [EHRs]). Despite some openness to features such as AI-driven alerts or remote monitoring, most clinicians emphasized the need for evidence of clinical use and a clear focus on reducing, not increasing, workload. Table 4 summarizes the key features and functionalities clinicians desire in a digital health solution, along with the main barriers and concerns, and indicates how many participants raised each point during the interviews.

**Table 3:** Desired features and functionality: the patient’s perspective (n=11).

Themes and subthemes		Prevalence, n (%)
**Diagnosis support and education**		
	Educational content tailored to women's heart health	10 (91)
	Summaries and videos explaining medications or symptoms	3 (27)
**Symptom awareness and early management**		
	Symptom logging	3 (27)
	Hormonal cycle insights	2 (18)
	Personalized alerts based on symptoms or wearable input	5 (45)
**Treatment management and adherence support**		
	Medication reminders	7 (64)
	Diet and lifestyle guidance	5 (45)
	Personalized health recommendations based on lifestyle and symptoms	5 (45)
	Appointment reminders	4 (36)
**Monitoring and tracking**		
	Vital signs tracking (BP^a^, HR^b^, ECG^c^, etc)	9 (82)
	Integration with wearables	9 (82)
	Sleep and stress tracking	3 (27)
	Cycle tracking	2 (18)
**Communication and feedback loops**		
	Real-time feedback or alerts when symptoms are concerning	8 (73)
	Automated report generation	6 (55)
	Ability to share data with HCPs^d^	6 (55)
	In-app messaging with the care team	4 (36)
**Motivation and well-being**		
	Exercise and mindfulness tips	2 (18)
	Community and patient support forums	2 (18)
**Barriers and concerns**		
	App complexity	4 (36)
	Privacy concerns	3 (27)
	Over-reliance or tech-induced anxiety	3 (27)
	Over-generalization of content	2 (18)
	No concerns	4 (36)

^a^BP: blood pressure.

^b^HR: heart rate.

^c^ECG: electrocardiogram.

^d^HCP: health care professional.

**Table 4:** Desired features and functionality: the clinician’s perspective (n=7).

Themes and subthemes		Prevalence, n (%)
**Treatment planning and customization**		
	Use of sex-specific guidance (pregnancy and menopause)	6 (86)
	Important to customize goals and treatment plans	6 (86)
**Monitoring preferences**		
	Prefer active patient monitoring	3 (43)
	Prefer alerts-only model	3 (43)
	Would not use the app for monitoring (education use only)	1 (14)
**Communication features**		
	Prefer automated patient reports	5 (71)
	In-app direct communication not preferred (prefer phone, email, and in-person follow-up)	5 (71)
**Alert preferences**		
	Prefer periodic summaries only	5 (71)
	Prefer both real-time alerts plus summaries	1 (14)
	Prefer alerts only for high-risk events	1 (14)
**Patient-generated data priorities**		
	Blood pressure readings	7 (100)
	Heart rate and rhythm	5 (71)
	Symptom tracking	4 (57)
	Medication adherence reports	4 (57)
	Hormonal cycle fluctuations	4 (57)
	Stress, sleep, and mental health indicators	3 (43)
	Exercise and activity levels	3 (43)
	Weight and BMI changes	2 (29)
	Lifestyle factors (eg, diet and smoking)	2 (29)
	CVD^a^ risk score integration	2 (29)
	Diagnostic data (eg, lab values and cholesterol)	2 (29)
**Barriers and concerns**		
	Excess workload and nonactionable data (noise)	4 (57)
	Data accuracy and false alarms	3 (43)
	Legal liability if alerts go unaddressed	3 (43)
	Poor EHR^b^ integration	3 (43)
	Privacy and data security concerns	3 (43)
	Lack of clinical use	2 (29)
	Patients’ over-reliance on the app	2 (29)
	Motivation and sustained engagement by patients	1 (14)

^a^CVD: cardiovascular disease.

^b^EHR: electronic health record.

Contrasting the two perspectives revealed strong alignment between patients and clinicians on the value of tracking vital signs, providing sex-specific guidance, and offering educational content tailored to women’s heart health. However, notable divergences emerged around communication, monitoring preferences, and the diagnostic support role of the app. Patients favored real-time feedback, interactive features, and personalized support, while clinicians expressed concerns about workload, data reliability, and legal liability, preferring structured summaries and limited app-mediated communication. These differences highlight the need for a dual-pathway design that balances patient empowerment with clinical workflow and safety.

Table 5 compares the perspectives of participating women with CVD versus cardiologists regarding desired app features across the patient journey. It highlights areas of alignment and divergence across different phases of the patient journey, and identifies where design focus is most needed to address gaps and support user-centered implementation.

**Table 5:** User requirements comparison across the cardiovascular disease patient journey (patients vs clinicians).

Feature cluster	Patient perspective	Clinician perspective	Alignment
Symptom onset and early risk tracking	Many patients want tools to log symptoms, track hormonal cycles, and receive alerts for serious changes.	Clinicians track symptoms but prefer periodic summaries over real-time alerts due to workload and liability concerns.	Partial
Diagnosis support	Patients often seek symptom explanations and hope for diagnostic guidance from the app.	Clinicians are wary of misdiagnosis; some see the app as educational but not diagnostic.	Divergent
Treatment adherence and medication	High interest in medication reminders, side effect information, and adherence support.	Clinicians value adherence tracking and side effect reporting if actionable, summarized, and integrated into their workflow.	Strong
Education and empowerment	Nearly all patients want tailored, female-focused educational content (eg, videos, articles, and lifestyle advice).	Clinicians agree that education is a key benefit of the app, especially if it supports better patient engagement.	Strong
Sex-specific and life stage guidance	All patients emphasized the need for advice linked to hormonal changes, pregnancy, and menopause.	Most clinicians strongly support sex-specific features to address overlooked risks and life-stage changes.	Strong
Vital signs and lifestyle monitoring	Most want to track vitals (BP^a^ and HR^b^) and sync with wearables for exercise, diet, and sleep insights.	Clinicians prioritize BP and HR and accept wearable data cautiously, questioning accuracy and clinical validity.	Moderate
Personalization and motivation	Strong interest in personalized advice and goal-setting features to support self-management.	Clinicians support customizable goals but are concerned about overburdening users or implying unsupported precision.	Partial
Communication with care teams	Many patients want in-app messaging, shared data, and reminders to feel more connected and supported.	Clinicians prefer structured reports and oppose app-based messaging due to time limits and workload.	Divergent
Monitoring preferences	Most prefer real-time feedback if needed, while some fear over-monitoring or anxiety from alerts.	The majority prefer summary reports; only a few support real-time data access or alerts, citing resource constraints.	Divergent
Trust, privacy, and usability	Simplicity and ease of use matter most; some express concerns about privacy and overalerting.	Clinicians worry about liability, data overload, and integration challenges; they prefer actionable, low-burden tools.	Moderate

^a^BP: blood pressure.

^b^HR: heart rate.

### Success Metrics and Willingness to Pay

Patients defined success with the app in terms of greater control, improved understanding, and emotional reassurance. Key success indicators include feeling more in control of their heart health (7/11, 64%), increased awareness of sex-specific symptoms and conditions (6/11, 55%), and reduced anxiety or stress (5/11, 45%), along with better adherence to medication and more personalized guidance. For many, success is also tied to the app’s ability to improve communication with health care providers and deliver trustworthy, up-to-date content.

Regarding willingness to pay, the average score was 7.1 on a scale of 1 to 10, with individual responses ranging from 3 to 10. Preferences for pricing models were mixed, with a slight preference for one-time payments (6/11, 55%), reflecting a desire for financial simplicity and predictability. Monthly models were preferred by 4 of 11 (36%) respondents for their flexibility, though some requested a free trial period as a prerequisite.

The primary factors influencing willingness to pay included the app's demonstrated usefulness, degree of personalization, sex- and age-specific features, trustworthiness, and perceived value for improving cardiovascular health. A few respondents flagged affordability and usability as potential barriers, highlighting the importance of designing an accessible and demonstrably beneficial tool. Table 6 provides an overview of patient-reported success metrics and expectations around payment for digital health solutions, including the number of participants who mentioned each aspect during the interviews.

**Table 6:** Patient-reported success metrics and payment expectations (n=11).

Themes and subthemes		Prevalence, n (%)
**Perceived success indicators**		
	Feeling more in control of heart health	7 (64)
	Increased awareness of sex-specific symptoms and conditions	6 (55)
	More personalized recommendations	5 (45)
	Reduced stress or anxiety	5 (45)
	Increased motivation to manage health	4 (36)
	Access to reliable and up-to-date health information	3 (27)
	Improved medication adherence	2 (18)
	Improved communication with doctors (eg, being taken seriously)	2 (18)
	Fewer emergency visits	1 (9)
**Preferred payment model**		
	One-time payment	6 (55)
	Monthly subscription	4 (36)
	Mixed (sees pros and cons in both)	1 (9)
**Payment decision drivers**		
	Proven usefulness and effectiveness	6 (55)
	Personalized recommendations or tracking	5 (45)
	Availability of sex- or age-specific features	4 (36)
	Free trial option	3 (27)
	Affordability or current financial situation	3 (27)
	Recommendation by a doctor or a trusted source	2 (18)
	Simplicity and ease of use	2 (18)
	Positive user reviews	1 (9)
	Direct communication or data sharing with HCP^a^	1 (9)

^a^HCP: health care professional.

Clinicians defined the success of a CVD app through its impact on patient engagement, adherence, and health outcomes, rather than direct clinical decision-making support. The most frequently cited indicators of success included improved patient engagement and participation in care management (6/7, 86%), greater adherence to treatment (5/7, 71%), and better patient education and symptom recognition (5/7, 71%), particularly regarding sex-specific cardiovascular risks. Several clinicians emphasized the need for such outcomes to be validated through research before adoption in routine practice.

Adoption incentives included features that support remote monitoring, patient education, and AI-supported risk assessments, provided they are accurate and well-integrated. Seamless integration into clinical workflows and EHRs, a simple interface, and support for research and data sharing were also valued.

Regarding willingness to pay, clinicians expressed moderate interest, with a mean score of 5.3 (SD 1.3) on a scale of 1 to 10. Preferences leaned toward monthly or yearly pricing models, with a desire for a free trial period. Willingness to pay was highly conditional on the app’s proven benefit to patient outcomes, usability, compliance with data protection, and ability to support research or institutional adoption. Table 7 provides an overview of clinician-reported success metrics and expectations around payment for digital health solutions, including the number of participants who mentioned each aspect during the interviews.

**Table 7:** Clinician-reported success metrics and payment expectations (n=7).

Themes and subthemes		Prevalence, n (%)
**Perceived success indicators**		
	Improved patient engagement and participation in care management	6 (86)
	Improved adherence to treatment	5 (71)
	Better symptom recognition and earlier referral	5 (71)
	Better patient education and self-management	5 (71)
	Reduction in preventable cardiovascular events or hospitalizations	3 (43)
	Must be tested in a research context	3 (43)
	Easier or faster appointments due to prefilled questionnaires	2 (29)
	Long-term lifestyle improvements	1 (14)
	Increased patient satisfaction and trust	1 (14)
**Must-have features for adoption**		
	Remote monitoring or real-time patient tracking	4 (57)
	Educational content (videos, disease explanations, etc.)	4 (57)
	AI^a^-driven risk assessments	3 (43)
	Ability to use data for research	3 (43)
	Patient engagement tools (behavior change and motivation)	3 (43)
	Simple, intuitive user interface	3 (43)
	Integration with EHR^b^ systems	3 (43)
**Preferred payment model**		
	Monthly payment	3 (43)
	Yearly payment (with trial preferred)	2 (29)
	One-time payment	1 (14)
	No preference	1 (14)
**Payment decision drivers**		
	Proven improvement in health outcomes and adherence	4 (57)
	Ability to support research (questionnaires and data export)	3 (43)
	Data security and privacy guarantees	2 (29)
	Institutional or guideline endorsement	2 (29)
	Direct benefit to patients (motivation and education)	2 (29)
	Ease of use (for both patients and providers)	2 (29)
	Broad adoption or market share	1 (14)
	Gender-specific features are fully integrated	1 (14)

^a^AI: artificial intelligence.

^b^EHR: electronic health record.

### Regulatory and Reimbursement Considerations

To complement the user requirements gathered from patients and clinicians, we conducted additional interviews with one regulatory expert (SN [nonauthor]) and one reimbursement expert (MF [nonauthor]), both with in-depth knowledge of the Swiss and broader European health care landscapes. This supplementary perspective is critical, as the app will be developed primarily for use in Switzerland, with a medium-term vision for deployment in other European contexts.

The regulatory expert assessed that an AI-enabled CVD app (AI-driven alerts and AI-supported risk assessments), designed to support women across the spectrum of symptom recognition, diagnosis, treatment adherence, and self-management, is likely to be considered a medical device under Swiss law. Specifically, if the app includes features such as AI-driven risk alerts, symptom tracking, and clinical report generation intended to support medical decision-making or influence health outcomes, it would fall under the Medical Device Ordinance as governed by Swissmedic. Depending on the final functionality, the app would likely be classified as a Class IIa medical device, requiring compliance with regulatory obligations related to safety, performance, and quality assurance.

Given that the app would likely use algorithms to generate health risk scores and may eventually integrate with electronic health records or wearable data for clinical review, transparency of its AI functionalities (eg, AI-driven alerts and AI-supported risk assessments) and proper documentation of its performance are essential. Although Switzerland is not part of the EU, it has harmonized its medical device regulatory system with the EU Medical Device Regulation (MDR). Therefore, developers must ensure conformity with Annex I of the MDR, particularly concerning software validation, cybersecurity, and human oversight of AI outputs. Early consultation with Swissmedic, including use of their pre-submission guidance services, was strongly recommended by the expert to confirm classification and identify the most appropriate pathway to conformity assessment.

From a data protection standpoint, the expert added that the app must comply with the Swiss Federal Act on Data Protection, which aligns closely with the EU’s General Data Protection Regulation. This includes obtaining explicit consent for processing health data, ensuring transparency in AI-based recommendations, and conducting a Data Protection Impact Assessment if sensitive data are used for profiling or personalized recommendations. Data must be stored securely in accordance with Swiss and European data security standards.

In terms of market access within Switzerland, the reimbursement expert explained that there is currently no established reimbursement pathway for digital health tools of this nature under the standard TARMED system (soon to be replaced by the new tariff system called TARDOC). However, several viable strategies exist. These include partnerships with supplemental insurance providers, integration into occupational health programs, or collaborative pilots with cantonal public health authorities focused on prevention and chronic disease management. Additionally, public-private partnerships involving academic hospitals could support real-world evidence generation, which will be critical for clinical validation and acceptance.

As the app evolves, modular feature deployment (eg, launching initially with educational content and lifestyle tracking before integrating AI-based alerts or EHR connectivity) may offer a lower-risk route to initial uptake. Positioning the app as an adjunct to care rather than a diagnostic tool can help mitigate medico-legal risks and facilitate clinician adoption. For future scaling into EU markets, preparation for CE (Conformité Européenne) marking and consideration of fast-track pathways like Germany’s Digitale Gesundheitsanwendungen (Digital Health Applications) process would be key strategic steps. A summary of regulatory and reimbursement considerations is provided in Multimedia Appendix 5, focusing primarily on Switzerland, with additional insights relevant to potential midterm expansion into the EU market.

## Discussion

### Primary Findings

This study offers detailed insights into how digital health tools can be designed to better serve women with CVD by directly addressing long-standing gaps in diagnosis, treatment, and self-management. What distinguishes this work is its grounding in sex-specific lived experiences and clinician workflows; most existing tools and studies either generalize across populations or fail to capture the unique challenges women face across the cardiovascular care continuum.

Our findings confirmed the diagnostic blind spots long documented in the literature but go a step further by detailing how these gaps are experienced by women, especially the emotional toll of feeling dismissed or misdiagnosed due to atypical symptom presentation, and how clinicians themselves acknowledge the limitations of current care pathways and tools. We also identified a mismatch between what patients expect from digital tools (eg, personalized, real-time, and educational support) and what clinicians view as feasible or desirable (eg, structured summaries and low-disruption integration), echoing broader implementation challenges in digital health but offering concrete, user-validated features to bridge this divide.

Furthermore, this work goes beyond user needs to surface actionable priorities for design, regulation, and reimbursement. By mapping unmet needs to specific app features and clarifying stakeholder requirements, we contribute an applied framework for the development of a digital tool that is not only patient-centered but also clinically and systemically grounded.

### Addressing Unmet Needs Across the Care Continuum

Our interviews with patients and clinicians confirmed the consistent disconnect between current cardiovascular care pathways and the lived experiences and needs of women with CVD. Both groups emphasized a clear gap in early recognition and diagnosis of CVD in women, often driven by atypical symptom presentation, a finding well-supported in the literature [4]. Women frequently report vague or nonspecific symptoms (eg, fatigue, anxiety, and back pain) that are either misattributed or dismissed, leading to delayed diagnosis and poorer outcomes. This aligns with clinical studies showing that atypical symptoms in women are associated with underdiagnosis and delayed care, a pattern historically described as the Yentl syndrome and reaffirmed in recent studies [27].

Clinicians similarly confirmed that CVD does not present in women as it does in men, often requiring multiple diagnostic steps and greater clinical vigilance. Yet, most participating clinicians admit to using standardized, male-centered diagnostic tools, with little to no adaptation for sex- or life-stage-specific risks (eg, menopause and pregnancy-related complications). This is consistent with research papers that highlight the lack of integration of sex-specific risk factors into clinical practice and guidelines [28,29].

Our findings also showed that there is a shared perception that awareness and education are insufficient, both for patients and providers. Patients often feel overwhelmed and underinformed, particularly about how hormonal changes might affect their heart health. Clinicians acknowledge this gap, noting that many are not trained to assess or communicate about sex-specific CVD risks, confirming concerns raised in the literature about persistent gender bias in cardiovascular medicine [30].

On the digital health front, while most patients have not used cardiovascular apps, citing lack of awareness, complexity, or emotional overwhelm, many express openness to tools that are personalized, educational, and easy to use. Importantly, all patients agreed that features like hormone-aware symptom tracking and cycle or menopause-specific guidance would be valuable. Clinicians were generally supportive of a digital tool, provided it reduces workload and offers actionable insights. However, many remain skeptical due to workflow integration concerns, unclear clinical use, and insufficient validation, echoing findings from other studies that cite similar adoption barriers [14,31-33].

This shows a strong concordance between user-reported unmet needs in our research and evidence in the scientific literature. The development of a CVD app specifically designed for women presents an opportunity to bridge the identified diagnostic support and management gaps by offering sex-specific decision support and longitudinal symptom tracking across life stages, in a way that integrates meaningfully into both patient journeys and clinician workflows.

### Balancing Patient-Centered Design With Clinical Workflow Integration

The synthesis of clinician and patient requirements revealed meaningful overlap in priorities for a CVD app tailored to women, particularly around core features such as BP, HR, cycle and symptom tracking, medication adherence tools, and educational content specific to women’s cardiovascular risks. Both groups also expressed interest in incorporating wearable data, lifestyle tracking, and AI-driven alerts, but with key differences in expectations around data flow and engagement. To ensure alignment with human factors (HF) principles, app features and interface components should be conceptualized to minimize cognitive load, support workflow compatibility, and accommodate differentiated engagement preferences between patients and clinicians.

Patients consistently emphasized the importance of holistic, personalized support, especially around lifestyle guidance (eg, diet and stress management), menstrual and hormonal tracking, and emotional well-being. Our recent research, which reviewed 20 commercially available CVD apps, showed that only 25% (5/20) offered sex-specific content, reinforcing that sex-specific app features in CVD are rare and needed [15]. Patients also valued real-time feedback and communication, but with flexibility to self-regulate interaction frequency, a finding supported by previous research [34].

Clinicians, by contrast, largely favored periodic summaries over real-time alerts, citing concerns about information overload, workflow disruption, and medico-legal liability, concerns echoed across several reviews on eHealth implementation challenges [14,32,33,35], underscoring the importance of minimizing cognitive workload and supporting efficient information processing in clinical settings. There was strong support for automated, concise reporting tools to enable faster clinical decision-making, particularly if data is aggregated and actionable (eg, medication nonadherence flags, high BP trends, and cycle fluctuation). Importantly, clinicians stressed the need for sex-specific clinical insights (eg, pregnancy-safe medication guidance), aligning with calls in the literature to address gender gaps in cardiovascular risk assessment and care pathways [36].

Despite agreement on many core features, divergence between patients’ and clinicians’ requirements remains. Clinicians were skeptical of communication via the app, preferring existing channels (email and phone), whereas many patients desired low-barrier communication tools or asynchronous Q&A features. Additionally, clinician trust in wearable data remains limited due to concerns about accuracy, despite evidence that wearables can support prevention strategies and risk monitoring when used appropriately [10,37].

These findings underscore the need to balance patient-centered design with clinical workflow integration, ensuring that features valued by patients (eg, education, personalization, symptom explanation, and risk prevention) do not impose undue burden on clinicians. The app should prioritize structured reporting, passive data aggregation, and modular engagement options, and be framed as an adjunct, not a replacement, for in-person care. Differentiated user models, with patient-facing tools focused on behavior change, motivation, and real-time support, and clinician-facing tools optimized for decision efficiency and minimal disruption, can support better task-technology fit for both groups. Interface design should adhere to HF principles such as customizability by role, transparency of AI outputs, clear visual hierarchies, and user autonomy in alert settings, all of which were strongly echoed in user preferences. Usability must remain a central design principle, interpreted not only as ease of use but also through HF constructs such as interaction efficiency, information clarity, and minimization of user burden. Successful uptake will hinge on transparent validation, privacy safeguards, and clinical evidence.

### Articulating Meaningful Success Narratives

The analysis of success metrics and willingness to pay highlighted distinct yet interrelated priorities between patients and clinicians that carry significant implications for the app’s value proposition and sustainable adoption. While both groups identify improved patient adherence, better education, and enhanced engagement as key indicators of effectiveness, they differ in how success is defined and operationalized.

Patients tend to frame success in terms of personal empowerment and perceived well-being, such as increased awareness of sex-specific symptoms and conditions, feeling in control of their heart health, reduced anxiety, and improved ability to interpret symptoms and make informed decisions. Most patients cited greater motivation and personalized insights as important outcomes, and several emphasized a desire for the app to help them feel taken seriously in clinical encounters. These findings align with research demonstrating that digital interventions can significantly boost patient empowerment, knowledge, and self-management in chronic illness [13,38]. This experiential framing of success suggests that patients value both functional benefits (eg, symptom recognition and adherence reminders) and emotional reassurance, and are likely to judge effectiveness through lived experience rather than clinical endpoints alone.

Clinicians, in contrast, emphasized evidence-based outcomes, such as reduced hospitalizations, earlier detection of complications, and measurable improvements in disease management. While some acknowledged softer benefits like improved communication or decreased workload due to better-informed patients, there was a consistent call for formal evaluation of impact, preferably through research trials, factors frequently noted in clinician acceptance studies [32,33]. This divergence highlights the importance of building a dual feedback system, one capturing patient-reported outcomes and engagement metrics, and another tracking clinical indicators that can be aggregated and validated over time.

In terms of willingness to pay, patients showed relatively high interest. Key influencing factors included the app’s ability to improve cardiovascular health, offer sex-specific and personalized recommendations, and provide reliable, easily digestible information, echoing user preferences reported in patients’ mHealth adoption literature [34]. Several patients noted the appeal of free trial options and transparent pricing, especially in light of economic constraints.

By contrast, clinicians expressed moderate willingness to pay and viewed app adoption as contingent on demonstrated clinical use, alignment with existing systems, and endorsement by institutions or guidelines. Most clinicians would expect the app to be provided at the health system or institutional level, particularly if it supports research or integrates with existing systems, factors shown as crucial for sustained clinician adoption in previous research [32,33,35].

Payment feasibility was primarily seen through the lens of institutional procurement rather than individual expenditure. This indicates that a tiered strategy could be considered, potentially offering direct-to-patient subscriptions for patients and licensing options or integration pathways for clinical and research settings. Such a dual-strategy may combine offering core low-risk features and premium add-ons directly to motivated patients, with an institutional offering that includes more advanced research data modules such as an AI-predictive model, EHR integration, and structured reporting and smart alerts. Free trials, outcome-based pricing, or inclusion in reimbursement schemes could improve uptake across both segments, as emphasized in similar research [39,40].

Overall, the findings suggest that to drive adoption and value across stakeholder groups, the app must articulate distinct success narratives, one rooted in empowerment and lived experience for patients, and one grounded in clinical efficiency and measurable outcomes for providers.

### Regulatory and Reimbursement Implications

Incorporating regulatory and reimbursement expertise early in the development process helps ensure that the solution is not only clinically and user-relevant but also aligned with the legal, safety, and financial frameworks required for real-world adoption. Failure to consider these aspects at an early stage often results in substantial implementation barriers and limits the scalability and sustainability of digital health innovations [14,32-35,41].

From a regulatory standpoint, the expert confirmed that if the app includes AI-driven functionalities, such as personalized risk alerts, clinical report generation, or features that support diagnosis or influence medical decision-making, it would likely qualify as a Class IIa medical device under Switzerland’s Medical Device Ordinance, harmonized with the EU MDR. This classification implies that the app must meet formal requirements related to software performance, cybersecurity, human oversight, and clinical evaluation, as outlined in MDR Annex I. Given the inclusion of health data processing and algorithmic personalization, the app must also comply with the Swiss Federal Act on Data Protection, which closely mirrors the general data protection regulation, including requirements for explicit user consent and transparency of algorithmic outputs. These recommendations align with recent regulatory reviews emphasizing the increased scrutiny of AI-based medical software and the importance of early consultation with regulatory bodies to validate classification and compliance pathways [42].

On the reimbursement side, the expert highlighted that Switzerland currently lacks a dedicated reimbursement pathway for digital health applications within the standard outpatient tariff system (TARMED), which is soon to be replaced by the new TARDOC system. In the midterm, TARDOC will be the key framework to monitor. According to the factsheet on digital health applications provided by the Federal Office of Public Health, digital tools, including AI applications, are expected to be classified as part of the infrastructure or personnel services [43]. This means they could potentially be reimbursed under specific TARDOC positions, such as those related to telemedicine. However, several alternative access models were identified, including partnerships with private health insurers, occupational health initiatives, and cantonal prevention programs. These strategies reflect recent calls in the literature for shifts in European digital health financing, which recognize the need for novel evidence-generation mechanisms to support the adoption and scaling of digital tools [44,45]. In Switzerland, reimbursement will likely depend on demonstrated value in real-world settings, particularly in enhancing adherence, promoting prevention, and reducing unnecessary health care use.

### Design Implications and Next Steps

To translate the diverse and specific user requirements identified in this study into actionable design features, we mapped patient and clinician needs along the cardiovascular care continuum. Thematic synthesis of interview data revealed critical gaps and opportunities at multiple points in the patient journey, from early symptom recognition to diagnosis support, treatment adherence, ongoing self-management, and broader contributions to cardiovascular research. These findings aligned with persistent shortcomings in the literature, particularly around under-recognition of sex-specific symptoms [46], insufficient tailoring of risk assessment tools, and limited integration of digital health tools into routine care for women with CVD [15].

**Figure 2:**
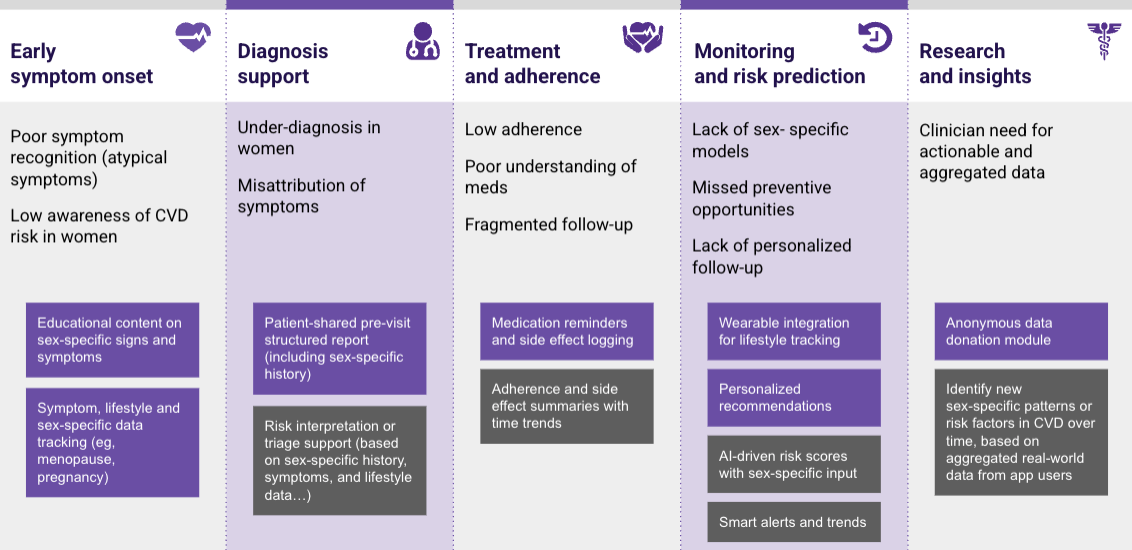
Design implications across the cardiovascular patient journey in women. AI: artificial intelligence; CVD: cardiovascular disease.

Figure 2 presents a high-level design framework illustrating how these needs will be addressed through core app features, organized by phase of care. Patient-facing features are shown in dark violet boxes and focus on supporting the lived experience of women navigating cardiovascular conditions, such as personalized education, symptom logging, and wearable-integrated tracking. Clinician-facing features, in the dark gray boxes in the figure, prioritize workflow efficiency, diagnostic support, and data integration. It’s also worth noting the interconnectedness of some of these elements. For example, the AI-driven risk alerts and clinical report generation depend on continuous, structured input from patient-generated data, including wearable metrics, symptom tracking, and medication adherence. Similarly, the research and insights feature, designed for clinician relevance, builds on aggregated app data to identify emerging sex-specific patterns and close gaps in clinical evidence.

This feature map provides a consolidated foundation for the next phase of development. It will serve as the blueprint for the initial prototype, which will undergo usability testing in an iterative design process, as highlighted in Figure 1 under the methodology section. Through repeated feedback loops with both patients and health care professionals, the prototype will be refined to ensure alignment with real-world needs and system-level constraints, ultimately enhancing adoption, clinical relevance, and long-term impact.

### Limitations and Future Research

This study was conducted exclusively in Switzerland, which may limit the generalizability of the findings to other health systems or cultural contexts. Although all patients were recruited via a single university hospital partner, the clinic serves a broad public population and includes individuals with varying levels of health and digital literacy. Nonetheless, this limitation will be addressed in the next phase of the project through iterative prototyping with a more demographically and geographically diverse patient sample. Given that the primary goal is to develop a tool for use in Switzerland, with longer-term plans to expand into other German-speaking countries such as Germany and Austria, we believe the study was appropriately scoped for this stage of development. Broader geographic and cultural representation will be integrated in the next phase of the development, particularly during prototype testing and validation.

The sample size was relatively small, in line with typical qualitative research. While this limits the breadth of perspectives captured, we achieved thematic saturation, suggesting that the findings are sufficiently robust to inform the current design phase. Nonetheless, some stakeholder groups were not represented in this study. In particular, general practitioners and nurses were not included due to recruitment challenges and resource constraints. Their perspectives, especially those of general practitioners, who often serve as the first point of contact during early symptom onset, are crucial for understanding barriers to timely recognition and referral. Future phases of the development will include these stakeholders to ensure that insights from across the care continuum are reflected in the final design.

### Conclusions

This study confirmed a persistent disconnect between existing cardiovascular care pathways and the specific needs of women living with CVD. Patients shared experiences of feeling underinformed, underheard, and underserved, particularly during early diagnosis support, due to atypical symptoms and a lack of sex-specific guidance. Clinicians acknowledged these challenges but admitted that current tools and workflows are still largely designed around male-centric risk models. Both groups agreed that digital health has the potential to bridge this gap but also highlighted different expectations; patients seek personalized education and empowerment, while clinicians prioritize clinical use, low burden, and workflow integration.

The participants’ input showed strong overlap in desired core features, such as sex-specific symptom and cycle tracking, medication adherence tools, and tailored educational content. However, divergences appeared around data communication and feedback. Patients favored more interactive, real-time support, while clinicians preferred summarized, actionable insights to avoid alert fatigue and liability concerns. These findings highlight the importance of modular, adaptable design, an app that offers flexibility in use, differentiated interfaces for patients and providers, and clearly defined roles for AI and automation.

These findings also inform key HF considerations for interface design. For clinicians, minimizing cognitive workload was critical; participants favored tools that provide actionable, low-effort insights rather than real-time data streams. Features such as passive data aggregation, structured dashboards, and summary reports were seen as more aligned with clinical workflows and information processing constraints. For patients, usability was tied to personalization, transparency, and flexible communication options. To ensure task-technology fit, the design must allow differentiated engagement based on user role, with modular, customizable features that minimize burden while maximizing relevance.

Success metrics also differed by perspective. Patients emphasized psychological and motivational benefits such as feeling in control, recognized, and informed, whereas clinicians focused on measurable outcomes such as adherence, hospital avoidance, and engagement. However, both agreed that success requires trust, usability, and relevance to daily life. These insights informed a dual-value strategy for app development, aligning patient empowerment with clinical efficiency.

Finally, our expert interviews highlighted that integrating regulatory and reimbursement perspectives early is key to a successful implementation. With features such as AI-supported insights and clinical data sharing, the app will likely qualify as a medical device under Swiss and European laws. Planning for staged implementation, starting with nonregulated educational features and expanding as validation and infrastructure grow, emerges as a practical strategy. However, long-term success and widespread adoption would largely depend on demonstrating real-world value and forming partnerships with insurers and public health programs.

## Appendix

Multimedia Appendix 1 Participant information sheet and consent form.

Multimedia Appendix 2 Sample characteristics of participating patients and clinicians.

Multimedia Appendix 3 Interview guides.

Multimedia Appendix 4 Thematic Analysis Process.

Multimedia Appendix 5 Summary of regulatory and reimbursement considerations.

## Abbreviations

AF: atrial fibrillation
AI: artificial intelligence
BP: blood pressure
CE: Conformité Européenne
CVD: cardiovascular disease
EHR: electronic health record
EU: European Union
HCD: human-centered design
HF: human factors
HR: heart rate
MDR: Medical Device Regulation
mHealth: mobile health
