# Supplementary material for: Bridging Gaps in Women’s Heart Health: User-Centered Needs Assessment Informed by Patient and Clinician Interviews
Source: JMIR Hum Factors. 2026 Jan 13;13:e82916. doi: 10.2196/82916 (PMC12848491; doi:10.2196/82916)
Supplement: Multimedia Appendix 1 [file humanfactors_v13i1e82916_app1.pdf]

Participant Information Sheet

User Requirements for a health app that better meets the unmet needs of women managing cardiovascular disease

Background: Cardiovascular disease (CVD) affects millions of people globally, yet many existing digital health solutions fail to address the specific needs of diverse patient populations, particularly women. Studies show that CVD symptoms often present differently in women, leading to challenges in diagnosis, treatment adherence, and disease management. Hormonal changes throughout life (such as during pregnancy, menopause, and menstrual cycles) can significantly impact heart health, yet most existing CVD tools and apps are designed with a one-size-fits-all approach that does not account for these differences.

This study aims to explore how a **mobile health application can better support** women in **managing their cardiovascular health**. By participating, you will contribute to the development of a tailored solution that meets the real-world needs of women with CVD, ensuring that their specific challenges and requirements are addressed in a way that current digital tools often fail to do.

Objective:
The University of Applied Sciences Northwestern Switzerland (FHNW) is conducting this study on behalf of the eHealth startup Vavken Health Labs to gather insights from patients and healthcare providers about their needs and requirements for a CVD app. The objective is to:

- **Identify user needs:** understand the core challenges that patients and clinicians face in cardiovascular health management, particularly among women.
- **Define key requirements:** Explore which features and functionalities should be integrated into the app to effectively support CVD management.
- **Understanding patient and clinician expectation:** Gain insights into what would make the app engaging, useful, and easy to integrate into daily health routine.

Your participation will help shape the final design of the application, ensuring it is user-friendly and meets the specific needs of **women managing CVD**.

Expected Outcomes: The interviews will provide detailed insights into the most important features, usability preferences, and barriers that must be addressed in the app's design. This feedback will directly inform the development of a prototype that aligns with patient and clinical needs, ensuring its effectiveness in real-world use.

Funding: This study is sponsored by Innosuisse (The Swiss Innovation Agency), grant number: 75940.1 INNO-ICT.

Participant Information FAQs

1. **Who can participate?**
   - Women aged 18 years or older
   - Diagnosed with cardiovascular disease (e.g., coronary artery disease, arrhythmia, etc.)
   - Have access to the internet and email
   - Comfortable using a teleconferencing tool (e.g., MS Teams) and willing to have their answers recorded

2. You can **refuse to take part** in this study without giving a reason

3. **Ethical approval:** The Ethics Committee of Northwest and Central Switzerland (EKNZ) reviewed this project information and determined that ethical approval was not required for this study according to the Federal Act on Research involving Human Beings, article 2 paragraph 1 (Req-2025-00183)

4. **What will happen to the results of the study?** The findings from this study will be used to enhance the app design and may be published in academic journals or presented at conferences

5. **What will I be asked to do?** Participants will take part in a50-60**-minute online interview**, where they will discuss their experiences with CVD management and share their requirements for a mobile health app that better addresses the needs of women with CVD.

6. **Your participation in the study will be anonymized**. Only the research team will have access to participants' data. No personal or identifiable data be included in the dissemination of the results, which will be aggregated and anonymized.

7. **Information that is collected from you** will be securely stored. Personal identifiable information (e.g. consent form) will be kept separately from the data. Participants will be assigned a study code number and identifying information stored separately from the data.

8. **Recording equipment** will be used to record the Interview session.

9. **You can withdraw from the study without giving a reason.** To do so please e-mail Christine.jacob@fhnw.ch and sangeetha-rose.puthanveettil@students.fhnw.ch. The last time it will be possible to withdraw your data is the **end of April 2025**, given it will not be possible to withdraw once the research analysis and write up has started or findings were published.

10. You do not have to answer any questions you do not wish to.

11. **Free app access**: the research budget won't allow for participant remuneration; however, the research team foresees a relevant social value in enhancing adherence and consequently intervention success once the app is launched. **Participants in the interview will have early and free access to the app once it is launched *(email required)*.**

12. If you have **any questions or complaints** about the study, please contact the main investigator at christine.jacob@fhnw.ch

Participant Consent Form

Title of the Study: *User Requirements for a health app that better meets the unmet needs of women managing CVD*

Principal Investigator: Dr. Christine Jacob christine.jacob@fhnw.ch

Lead researcher: Sangeetha-Rose Puthanveettil sangeetha-rose.puthanveettil@students.fhnw.ch

1. I agree to take part in the above research. I have read the Participant Information Sheet (Version 1.1 - March 2025) for the study. I understand what my role will be in this research and all my questions have been answered to my satisfaction.
2. I understand that I am free to withdraw from the research at any time until the end of April 30th, 2025, without giving a reason.
3. I am free to ask any questions at any time before and during the study.
4. I understand what will happen to the data collected from me for the research.
5. I have been provided with a copy of the Participant Information Sheet.
6. I understand that non-identifiable quotes from me could be used in the dissemination of the research, and I am expressing my personal views.
7. I understand that the testing session will be recorded.

Data Protection and Ethical Approval: I agree to the University[1] processing personal data, which I have supplied. I agree to the processing of such data for any purposes connected with the Research Project as outlined to me*. Recordings will be retained for a period of 12 months and deleted afterwards.

The Ethics Committee of Northwest and Central Switzerland (EKNZ) determined that ethical approval was not needed for this study according to the Federal Act on Research involving Human Beings, article 2 paragraph 1 (reference number Req-2025-00183).

Name of participant ....

Email ....

Phone number ....

Place / Date ....

Signature ....

I WISH TO WITHDRAW FROM THIS STUDY

If you wish to withdraw from the research, please speak to the researcher or email them at christine.jacob@fhnw.ch stating the title of the research. You do not have to give a reason for why you would like to withdraw. Please let the researcher know whether you are/are not happy for them to use any data from you collected to date in the write up and dissemination of the research.

Participant Identification Number for this study: ....

[1] "The University" refers to the University of Applied Sciences Northwestern Switzerland (FHNW)

Innovation cheque supported by

Schweizerische Eidgenossenschaft
Confédération suisse
Confederazione Svizzera
Confederaziun svizra

Swiss Confederation

Innosuisse – Swiss Innovation Agency

VAVKEN HEALTH LABS

n|w Fachhochschule Nordwestschweiz
