# Supplementary material for: Bridging Gaps in Women’s Heart Health: User-Centered Needs Assessment Informed by Patient and Clinician Interviews
Source: JMIR Hum Factors. 2026 Jan 13;13:e82916. doi: 10.2196/82916 (PMC12848491; doi:10.2196/82916)
Supplement: Multimedia Appendix 2 [file humanfactors_v13i1e82916_app2.pdf]

Sample characteristics of participating patients (N=11)

Demographics and characteristics		Values, n (%)
**Sex**		
	Female	11 (100)
**Location**		
	Switzerland	11 (100)
**Age group**		
	35-44	1 (9)
	45-54	1 (9)
	55-64	4 (36)
	65+	5 (45)
**When they were first diagnosed with CVD**		
	Less than 1 year	3 (27)
	1-3 years	3 (27)
	8-10 years	2 (18)
	10+ years	3 (27)

Sample characteristics of participating clinicians (N=7)

Demographics and characteristics		Values, n (%)
**Sex**		
	Female	3 (43)
	Male	4 (57)
**Location**		
	Switzerland	7 (100)
**Age group**		
	35-44	1 (14)
	45-54	4 (57)
	55-64	2 (29)
**Affiliation**		
	University Hospital	3 (43)
	Cantonal Hospital	1 (14)
	Private Clinic	3 (43)
**How many years they have been treating patients with CVD**		
	10+ years	7 (100)
