# Supplementary material for: Bridging Gaps in Women’s Heart Health: User-Centered Needs Assessment Informed by Patient and Clinician Interviews
Source: JMIR Hum Factors. 2026 Jan 13;13:e82916. doi: 10.2196/82916 (PMC12848491; doi:10.2196/82916)
Supplement: Multimedia Appendix 3 [file humanfactors_v13i1e82916_app3.pdf]

Interview Guides

User Requirements for an app that better meets the unmet needs of women managing CVD

Opening message for both interviews (to provide context)

"Cardiovascular disease (CVD) presents differently in women than in men, with symptoms that are often under-recognized, misattributed, or overlooked. Hormonal changes throughout life (such as during pregnancy, menopause, and menstrual cycles) can significantly impact heart health, yet most existing CVD tools and apps are designed with a one-size-fits-all approach that does not address these differences. We're exploring how a more tailored solution could better meet the needs of women managing CVD, and we'd love your insights to help shape an app that truly reflects their experiences."

Patients' Interview

Focus: Usability, motivation, and alignment with the patient journey (especially for women's CVD needs).

Background Questions

- Gender
- Age
- When were you first diagnosed with CVD
- Clinic *(we will have this information, we won't need to ask them)*

Understanding the Patient Journey

1. Can you describe your experience managing your CVD? Have HCPs addressed how life stages like pregnancy, menopause, or hormonal changes impact your heart health?
2. What challenges do you face in monitoring your CVD or following your treatment plan? Do you feel current treatment pathways and tools consider hormonal influences on CVD?

Daily Use and Lifestyle Fit

3. Have you used any apps for CVD? If yes, which ones and what was your experience? If not, what has kept you from using them?
4. What features do you find most useful - or would you, if you haven't used any apps before? Would gender-specific tracking (e.g., hormonal changes, pregnancy, menopause) be beneficial?
5. Have you faced any challenges using health apps? (e.g., lack of personalization, male-centered design, complexity, data security concerns)

Features and Functionality

6. What features would be most helpful for managing your CVD? (e.g., tracking vitals, symptom logging, hormonal cycle insights, educational content tailored to women)
7. Would you like the app to provide sex specific personalized recommendations for diet, exercise, stress management, or medication?
8. How important is it for the app to sync with wearable devices (e.g., smartwatches, fitness trackers) and track menstrual cycle variations alongside cardiovascular metrics?

Communication and Support

9. How should the app help you communicate with your doctor or care team? Would sex-specific reports or AI-driven alerts for symptoms be useful?
10. Would you like the app to provide real-time feedback or connect you to a healthcare provider when issues arise?

Barriers and Concerns

11. What concerns might prevent you from using the app? (e.g., privacy, lack of trust in health tech, difficulty navigating apps, lack of gender-specific insights, overly generalized recommendations)

Success Metrics & Willingness to Pay

12. What would success look like for you when using this app? How would you know it's helping you improve your cardiovascular health?
13. If this app effectively meets your needs, how likely would you be to pay for it?
14. What price range would you consider reasonable (monthly vs. one-time)?
15. What factors would influence your willingness to pay?

HCPs' Interview

Focus: Workflow integration, usability, and clinical effectiveness, with emphasis on addressing unmet needs in women's CVD care.

Background Questions

- Gender
- Age
- How many years have you been treating CVD patients
- Clinic *(we will have this information, we won't need to ask them)*

Understanding Clinical Context

1. What challenges do you face in diagnosing and managing CVD? Do you find that women's symptoms may be overlooked or misattributed compared to men's?
2. How do you currently track and monitor CVD progression in your female patients, and does this differ from how you approach male patients?
3. Have you recommended any CVD apps to your patients? (If yes, which ones and why? If no, why not?)
4. What are the biggest strengths and weaknesses of existing CVD apps? Do they adequately address sex-specific symptoms, hormonal fluctuations, or life-stage changes like pregnancy and menopause?

Workflow Integration

5. What technologies (e.g., EHRs, remote monitoring tools) do you currently use in your workflow?
6. How could a CVD app best integrate with your existing systems or processes? (e.g. Would automatic data syncing with EHRs or patient reports improve usability?)
7. At what points in your workflow would additional data or decision support for women's CVD management be most beneficial? (e.g., tracking pregnancy-related cardiac risks, menopause-related changes, early warning alerts for sex-specific symptoms)

Features and Functionality

8. What types of patient data would be most valuable for you to receive from the app? (e.g., blood pressure, heart rate, symptom tracking, medication adherence, hormonal cycle fluctuations)
9. Would real-time alerts for critical issues (e.g., high-risk symptoms, medication non-adherence) be helpful, or would you prefer periodic summaries/reports?
10. How important is the ability to customize patient goals or treatment plans within the app? Would sex-specific guidance, such as pregnancy-safe recommendations or menopause-related risk adjustments, be useful?

Communication and Collaboration

11. How should the app facilitate communication between you and your patients? Would automated reports, in-app messaging, or alerts for critical changes be preferable?
12. Would you prefer active patient monitoring (where you can access real-time data) or a system that only notifies you of critical concerns? Or both?

Barriers and Concerns

13. What concerns might you have about using such an app? (e.g., data accuracy, liability, patient adherence, integration challenges)
14. Are there specific features that would make you more likely to adopt the app in your practice? (e.g., clinical decision support, AI-driven risk assessments, personalized treatment recommendations)

Success Metrics & Willingness to Pay

15. How would you measure the success or effectiveness of this app in your practice? (e.g., improved patient adherence, earlier detection of complications, better patient engagement)
16. What outcomes would indicate that the app is improving care for your female CVD patients? (e.g., better symptom recognition, reduced misdiagnosis, improved management through different life stages)
17. If this app effectively meets your needs, how likely would you be to pay for it?
18. What pricing model (monthly vs. one-time) would be more viable, and what factors would influence your willingness to pay?
