# Supplementary material for: Bridging Gaps in Women’s Heart Health: User-Centered Needs Assessment Informed by Patient and Clinician Interviews
Source: JMIR Hum Factors. 2026 Jan 13;13:e82916. doi: 10.2196/82916 (PMC12848491; doi:10.2196/82916)
Supplement: Multimedia Appendix 4 [file humanfactors_v13i1e82916_app4.pdf]

Multimedia Appendix 4: Thematic Analysis Process

This study employed the thematic analysis framework by Braun and Clarke [1,2] to guide the systematic coding and theme development process. Below is a summary of the analytic steps applied:

Familiarization with the Data
All interviews were audio-recorded, transcribed, and read multiple times by the researchers to ensure familiarity with the content. Initial notes and reflections were documented during this phase to begin sensitizing the analysts to emerging patterns.

Generating Initial Codes
A preliminary coding framework was developed based on the research questions and prior work by the team on cardiovascular digital health. The patient and clinician transcripts were initially coded by SP, while the regulatory and reimbursement interviews were coded by CJ. Coding was done using NVivo, and codes were assigned both deductively (based on the research questions and prior work by the team on cardiovascular digital health) and inductively (as they emerged).

Searching for Themes
Codes were reviewed to identify potential themes and subthemes. Related codes were grouped and organized into broader thematic categories aligned with the study's objectives (e.g., symptom recognition, sex-specific gaps, usability preferences, system integration barriers).

Reviewing Themes
Themes were reviewed for internal consistency. This involved iterative discussions between SP and CJ, with validation by PV in cases of disagreement. Coding discrepancies and theme boundaries were resolved through consensus-building discussions to ensure analytic rigor.

Defining and Naming Themes
Each theme was carefully defined to reflect the underlying data and assigned a descriptive name that captured its essence. Themes were mapped to phases of the care continuum where applicable (e.g., early diagnosis, treatment, long-term management).

Producing the Report
The finalized themes and subthemes were presented in the Results section. The synthesis guided the design implications and served as the foundation for generating user/system requirements and regulatory inputs.

1. Braun V, Clarke V. Successful Qualitative Research: A Practical Guide For Beginners. SAGE Publications Ltd; 2013. doi: 10.1002/jmr.2361
2. Braun V, Clarke V. What can "thematic analysis" offer health and wellbeing researchers? Int J Qual Stud Health Well-being 2014 Jan 15;9(1):26152. doi: 10.3402/qhw.v9.26152
