# Supplementary material for: Bridging Gaps in Women’s Heart Health: User-Centered Needs Assessment Informed by Patient and Clinician Interviews
Source: JMIR Hum Factors. 2026 Jan 13;13:e82916. doi: 10.2196/82916 (PMC12848491; doi:10.2196/82916)
Supplement: Multimedia Appendix 5 [file humanfactors_v13i1e82916_app5.pdf]

Multimedia Appendix 5: Summary of regulatory and reimbursement considerations

Domain	Switzerland (Primary Focus)	EU (Mid-term Expansion)
Medical Device Status	Likely classified as a Class IIa medical device under MedDO, due to AI risk alerts, decision support, and monitoring.	Equivalent classification under EU MDR. Must meet Annex I General Safety and Performance Requirements.
Regulatory Authority	Swissmedic	National Competent Authorities (e.g., BfArM in Germany, ANSM in France), coordinated under EU MDR.
Software Compliance	Must follow ISO 13485, ISO 14971, and relevant software lifecycle standards (e.g., IEC 62304, IEC 82304-1).	Same standards and cybersecurity controls apply. For AI, also ensure transparency, human oversight.
AI Oversight	Explainability, auditability, and clinical oversight required, especially for AI-driven features.	Align with EU AI Act. High-risk classification likely; must ensure human-in-the-loop design.
Data Privacy	Must comply with Swiss FADP. Explicit consent, transparency of data use, DPIA required for sensitive profiling.	Must comply with GDPR. Similar obligations apply, with emphasis on AI transparency and data minimization.
Clinical Validation	Real-world evidence needed for claims. Post-market surveillance mandatory. Pilot studies with hospitals recommended.	Clinical investigations required for CE mark (for higher-risk functionalities). Data reuse from Swiss studies possible.
Reimbursement	No standard reimbursement yet. Consider supplemental insurance partnerships and pilot programs.	Explore DiGA (Germany), PECAN (France), and regional pilots as pathways to reimbursement and listing.
Liability and Safety	Must clarify clinical responsibility boundaries. App framed as a decision aid, not a replacement.	Similar concerns apply. Must manage medico-legal risk through disclaimers and defined clinical workflows.
